# The Essential Tremors: Evolving Concepts of a Family of Diseases

**DOI:** 10.3389/fneur.2021.650601

**Published:** 2021-03-26

**Authors:** Elan D. Louis

**Affiliations:** Department of Neurology, University of Texas Southwestern, Dallas, TX, United States

**Keywords:** essential tremor, the essential tremors, terminology, classification, heterogeneity, disease

## Abstract

The past 10 years has seen a remarkable advance in our understanding of the disease traditionally referred to as “essential tremor” (ET). First, the clinical phenotype of ET has been expanded from that of a bland, unidimensional, and monosymptomatic entity to one with a host of heterogeneous features. These features include a broader and more nuanced collection of tremors, non-tremor motor features (e.g., gait abnormalities) and a range of non-motor features, including cognitive, psychiatric, sleep, and other abnormalities. The natural history of these features, as well as their relationships with one another and with disease duration and severity, are better appreciated than they were previously. Studies of disease etiology have identified a number of candidate genes as well as explored several environmental determinants of disease. In addition, the decade has seen the beginnings and expansion of rigorous postmortem studies that have identified and described the postmortem changes in the brains of patients with ET. This emerging science has given rise to a new notion that the disease, in many cases, is one of cerebellar system degeneration. Across all of these studies (clinical, etiological, and pathophysiological) is the observation that there is heterogeneity across patients and that “essential tremor” is likely not a single disease but, rather, a family of diseases. The time has come to use the more appropriate terminology, “the essential tremors,” to fully describe and encapsulate what is now apparent. In this paper, the author will review the clinical, etiological, and pathophysiological findings, referred to above, and make the argument that the terminology should evolve to reflect advances in science and that “the essential tremors” is a more scientifically appropriate term.

## Introduction

During the last decade or two, we have witnessed notable advances in our understanding of the neurological disease that traditionally has been referred to as “essential tremor” (ET). Advances have spanned several key areas, from clinical features to natural history and from etiology to disease pathogenesis. This evolution is largely driven by new data and, along with these new data, a growing appreciation of the broader diversity and assortment of clinical features, etiological factors, and pathophysiological mechanisms. In this paper, we review the clinical, etiological, and pathophysiological heterogeneity in ET and put forth the argument that the terminology should adapt to reflect advances in science and that “the essential tremors” is a more scientifically appropriate term.

## Expansion of the Clinical Phenotype of ET

### Introduction

The clinical phenotype of ET has expanded from that of a bland, unidimensional, monosymptomatic entity to one with a diverse array of features. These may include both a broader and a more nuanced assemblage of tremors, the appearance of motor features aside from tremors (e.g., gait abnormalities), and a range of non-motor features, including cognitive, psychiatric, and sleep abnormalities, among others. Here, we review the details.

### Tremors

A myriad of tremors may be seen in patients with ET. The primary clinical feature of ET is kinetic tremor (1–4). This may be observed during a range of activities of daily living, from writing to drinking to eating, and may be elicited on neurological examination during a variety of maneuvers (e.g., finger-nose-finger maneuver, spiral drawing, pouring water between two cups) (1, 5). In ~50% of ET patients, the tremor has an intentional component (6), with observed worsening of tremor as the patient approaches the target (i.e., either the finger or the nose) during the finger-nose-finger maneuver. Intention tremor in ET is not limited to the arms; 10% of ET patients exhibit intention tremor in their neck when their head approaches a target (7). This may be observed, for example, when the patient lowers their head to meet the cup or spoon as it approaches their face during the tasks of drinking or using a spoon (1). Intention tremor is observed during toe-to-target movements in 27.3% of ET patients (8).

In addition to kinetic and intention tremors, patients with ET often have postural tremor of the arms, which can range in severity, although the amplitude of this tremor is generally lower than that of the kinetic tremor (3, 9).

Tremor at rest, without the other cardinal features of Parkinsonism such as bradykinesia or rigidity, occurs in ~1–35% of patients with ET, depending on the method of case ascertainment (10, 11). In contrast to that seen in patients with Parkinson's disease, it is a late-disease feature, and it has only been observed in the arm (i.e., it has not been observed in the leg) (1, 2, 10, 11).

Over time, there is a tendency for the tremor in ET to involve other body regions aside from the upper limbs, and patients may develop cranial tremors, involving the neck, voice, or jaw (1, 12, 13). Hence, there is heterogeneity not only with respect to the activation condition during which tremor is observed (e.g., kinetic, postural, intention, and rest) but with respect to the somatotopic distribution of tremor. Cranial tremors, and especially neck tremor, is particularly prevalent in women with ET, among whom the prevalence of neck tremor is several times higher than that of neck tremor in men with ET (14, 15). This neck tremor often begins as a uni-directional tremor, either “no–no” (i.e., horizontal) or “yes–yes” (i.e., vertical); with time this can evolve into a more complex, multi-directional tremor (1, 16).

### Other Motor Features

The motor features of ET are not limited to tremors. Another motor feature of ET is gait ataxia (17–19), which may be elicited on neurological examination by asking patients to perform tandem gait. The number of tandem gait missteps in ET is in excess of that seen in control subjects of similar age (17, 19). In most ET patients, this ataxia is mild, although in some ET patients it may reach moderate severity (20). This ataxia has been shown to result in a reduction in patients' confidence in balance and a mild but significant increase in the number of near-falls and falls in ET patients compared to age-matched controls (21). There are several studies that suggest that certain phenotypic features (e.g., midline tremor) track with greater gait difficulty (22, 23). Subclinical eye movement abnormalities (24–26) as well as other motor abnormalities (e.g., eye-hand incoordination, greater temporal variability in repetitive movements, and abnormalities in motor learning) point to what is likely to be a more pervasive underlying abnormality of cerebellar function in ET (27–31).

There are other motor features as well. Several studies have also reported the presence of movement slowness in finger tapping and other tasks in ET cases compared to controls, with a heterogeneous range of values across ET cases (32–34).

The above discussion would be incomplete without a discussion of dystonia. There is growing recognition and acceptance that some degree of dystonia may be present during the examination of patients with ET (35–38). It is apparent that the presence vs. absence, distribution, severity, and natural history of that dystonia is not uniformly distributed across patients, although further work needs to be done to define the full spectrum of dystonic postures in patients with ET. Such work will have obvious implications for the conceptualization and framing of the entity now referred to as “dystonic tremor.” The ensuing discussion should recognize that different underlying disease entities may have overlapping clinical features; in this case, both tremor and dystonia might be referable to a disordered cerebellum (39–41).

### Non-motor Features

As with many neurodegenerative movement disorders (e.g., Parkinson's disease, Huntington's disease), the clinical features in ET extend beyond the motor system. These non-motor features may be divided into those that are cognitive, psychiatric, sensory, and other (e.g., sleep). These have been reviewed in detail elsewhere (42–47).

Beginning with studies published nearly two decades ago, investigators observed mild cognitive deficits in patients with ET when compared with controls, and the number of such studies is considerable (42, 48, 49). These deficits involve a number of cognitive domains, particularly executive function and memory (50). Studies have documented that the rate of cognitive decline in older ET patients is greater than that observed in age-matched controls (51). Epidemiological studies in Spain and New York have demonstrated that, beyond the presence of mild cognitive deficits, ET is associated with both an increased odds of prevalent dementia (52, 53) and an increased risk of incident dementia (53, 54). Conversion rate in ET from mild cognitive impairment to dementia seems to be in excess of that seen in control groups (55). The basis for the cognitive changes and dementia in ET is likely to be multi-factorial, and further studies are needed (42, 56, 57).

Many neurodegenerative diseases are indeed neuropsychiatric disorders. In ET, the presence of specific personality traits has been demonstrated in several studies (58–60), as well as a range of psychiatric features (anxiety, social phobia, and depression) (61–63), and there is evidence that some of these (e.g., depression) could be primary rather than a response to the disabling features of tremor (64).

Olfactory deficits have been variably reported in some although not all ET cohorts (65, 66), and hearing deficits have more consistently been reported in other cohorts (67–69). Sleep abnormalities have consistently been demonstrated in patients with ET (70–72).

### Additional Clinical Features

The age of onset in ET is not uniform. That is, there is considerable heterogeneity. Whether an individual who develops the disease at age 40 years has the same underlying disease as someone who develops the disease at a more advanced age (e.g., 75 years) is an interesting question. To date, no compelling data have been presented to suggest that there is an age cutoff for developing ET. In a similar sense, there is no age cutoff for Parkinson's disease or Alzheimer's disease.

### Electrophysiological Features

Electrophysiological studies also point to heterogeneity in ET. For example, there is evidence from kinematic recordings that ET cases with head tremor differ from those without head tremor with respect to the severity of their limb tremor (73). Other electrophysiological studies, using electromyography and testing of long-latency reflexes, suggest that ET cases are dividable into distinct groups based on the mode of activation of antagonist muscles or reflex pattern (74, 75). Different responses to cerebellar transcranial magnetic stimulation, observed across studies, could also be the result of heterogeneity across patient groups (76, 77).

### Neuroimaging Features

A variety of neuroimaging studies in ET have attempted to identify subdivisions of patients who differ with respect to neuroimaging features. There is some evidence that patients with head tremor differ from those without head tremor in resting-state fMRI studies (78) and that in tractography studies, ET patients with vs. without resting tremor differ from one another with respect to structural connectivity (79).

### Pharmacological and Surgical Response Phenotype

Additional evidence of heterogeneity in ET comes from the observed variable response to medications across patients. It is a common observation in clinical trials that responsiveness to medication is not uniform across patients and that there tend to be responders and non-responders and that the proportion of the latter is sizable (80). Several studies have shown that patients with specific phenotypic, electrophysiologic, or neuroimaging features respond more favorably to propranolol (74, 81).

There is also some evidence in the literature that surgical responsiveness may differ across ET patients with, for example, thalamotomy used as a salvage solution in patients who do not respond to deep brain stimulation surgery (82). However, one study that examined clinical correlates of deep brain stimulation surgical outcome across ET patients did not identify any clinical characteristics that correlated with response (83).

### Summary

The past decade or two has seen an expansion of the ET phenotype. This is broadly recognized in the field. How to deal with this heterogeneity is not clear. There have been some initial attempts to develop new nomenclature to acknowledge that ET might not comprise a single entity (e.g., ET vs. “ET-plus”) (38), although the proposed terminology has been criticized, and further work is needed (37, 84–87). More specifically, it is important to recognize clinical heterogeneity within ET; however, it is then important to take additional steps to determine whether that clinical heterogeneity is a marker of distinct, separable underlying etiological, pathophysiological, and/or mechanistic entities. If the clinical differences are not linkable to such meaningful differences, then they are superficial ones, and they should not be used as the basis for decisions about disease classification and disease nomenclature.

## Greater Understanding of the Natural History of ET and Recognition of Heterogeneity Across Patients

Over the past decade or two, we have developed a greater understanding of the natural history of ET. In most individuals with ET, tremor amplitude increases with time (88, 89). The pattern of progression is not the same in all individuals. Several patterns of progression have been described, the two most common of which are (1) late life onset (i.e., after age 60) with progression and (2) early life onset (i.e., before age 40) with mild, stable tremor for many years followed in the 60s and onwards with progression (1). The least common pattern is that of early life (e.g., childhood) onset with marked worsening over the ensuing decade (1).

Aside from the above-described heterogeneity in *pattern* of progression, we also know that patients are not homogeneous with respect to *rate* of progression. There are faster progressors and slower progressors (90, 91), and in ET families, there is a fourfold difference in rate of progression, with some families being markedly faster progressors than others (92). A number of factors have been identified that seem to track with or predict differences in rate of progression, including older age of onset (90), family membership (slower vs. faster progressing family) (92), and asymmetrical tremor (91).

A feature of progression in ET is the layering on of additional tremors with time [e.g., intention tremor (6), rest tremor (11), and head tremor (15)], with the number of such features increasing over time (93). Yet these features do not monotonously each appear in all ET patients—patients differ with respect to their development of these features.

In this discussion, we have focused on motor features of ET and specifically tremor. Yet there is evidence that non-motor features, and particularly cognitive deficits, occur in ET. Some patients go on to develop mild cognitive impairment or dementia; however, the development of these more severe forms of cognitive impairment are not uniform; some patients dement and others do not (55).

From the above discussion, one may see that on multiple planes, there is heterogeneity across patients with respect to natural history, with differences in pattern of progression, rate of progression, and features of progression.

## Advances in Knowledge Regarding Disease Etiology and Recognition of Etiological Heterogeneity

A number of genes have been associated with familial ET, with these genes either present in a single family or a small number of families (94, 95). Genome-wide association studies have found associations between ET and single nucleotide polymorphisms (SNPs) in the region of LINGO1, and other such studies have identified SNPs in other genes (STK32B, PPARGC1A, and CTNNA3) (95). What is apparent is that a host of genetic factors is likely to be linked with ET and that there is genetic heterogeneity (96, 97). Stated another way, there is more than one genetic cause for ET. Furthermore, there are environmental determinants of disease as well (98, 99), indicating additional etiological heterogeneity.

## Greater Understanding of Underlying Disease Pathophysiology and Recognition of Pathophysiological Heterogeneity in ET

The decade has seen the beginnings and expansion of rigorous and controlled postmortem studies of ET brains, and these have identified and systematically cataloged the postmortem changes in the brains of patients with ET. This new science has given rise to a new notion that the disease, in many cases, is one of cerebellar system degeneration (100–104). This being said, there is evidence of heterogeneity. While the majority of ET cases evidence a host of related degenerative features in the cerebellar cortex (105), a sizable minority of cases has Lewy body pathology (105), and an even smaller number has intranuclear inclusions (106, 107). These data indicate that the postmortem findings, and hence the pathomechanisms, are not uniform across all ET cases. Even within ET cases with cerebellar pathology, there is a range of severity of such pathology (102, 105).

## Heterogeneity Across a Continuum—What Is a Family of Diseases?

The modern concept of disease is that etiological factors (i.e., genetic or environmental) are the proximate causes of disease, and these set a series of pathophysiological processes in motion, which then result in clinical features. Hence, in terms of timed events, etiology leads to pathophysiology, and this in turn leads to clinical features. As such, a “disease” is defined by its features along a time continuum, spanning from etiology to pathophysiology to clinical.

We observed from the above discussions that all along this continuum, there is evidence of multi-dimensionality, variety, and diverseness, that is, heterogeneity. It is likely that specific elements in the proximate cause of ET (etiology) will eventually be linkable to particular elements in pathophysiology and in clinical features—in other words, that certain causes will be linked with certain pathophysiologies and this, in turn, with a specific constellation of clinical features.

This is not revolutionary thinking. In the same way, during the second half of the 20th century, it became apparent that not all of the Parkinsonisms were the same—that progressive supranuclear palsy, for example, was distinguishable on each of these planes (different genes, different postmortem findings, and overlapping but differing clinical features) from idiopathic Parkinson's disease. Similarly, “motor neuron disease” encapsulates a discrete set of diseases within this broad disease family. Parkinsonisms, motor neuron disease—these are *families of diseases*. In ET, current knowledge of genetic causes and pathophysiology are quite rudimentary, making it difficult at this juncture to define etiological-pathophysiological-clinical entities (i.e., “diseases”) that exist within “the essential tremors,” but it is only a matter of time before such links are observed. Preliminary work suggest that certain anatomic features of ET are linkable to pharmacological response phenotype, for example (81), and that certain clinical features (i.e., older onset) are associated with more degenerative pathology in ET (90).

## A Call for More Appropriate Terminology That Matches Our Understanding

In science, one may reach the point when the existing terminology is lagging behind the state of knowledge. While some degree of disconnect is not problematic, when it reaches a point when the terminology is incorrectly framing and falsely characterizing the entity it is meant to apply to and when it interferes with clear thinking about the entity, it is time for a change. “Essential tremor” is in fact a term that was coined in the second half of the 19th century (108); this was a different time. Based on the arguments put forth in this paper, it is now time to recognize that we are dealing with a family of diseases, more appropriately referred to as “the essential tremors.” While it may seem premature to start to use this terminology in the absence of a clear knowledge of the different diseases, there is enough evidence of heterogeneity that the terminology should change in advance, as the science is certainly heading in this direction. Indeed, in this paper, data are presented from more than 100 peer-reviewed studies, which support the thesis that ET is not one entity and is therefore more than one entity—the ETs. The term “ET-plus” was coined in acknowledgment of this heterogeneity, although differentiation solely on clinical features is overly simplistic, and this terminology should not stick. Conceptually, however, that attempt to acknowledge heterogeneity and to modify and broaden terminology is an acknowledgment that ET is a family of diseases.

One may ask whether it might not be better to preserve the term “ET” for those cases with a limited and classical set of clinical features and separate out other cases who have additional features. This is a problematic approach. We know from genetic studies and postmortem studies that ET cases with different etiologies and different pathophysiologies can share the same clinical phenotype; hence, there is etiological and pathophysiological heterogeneity (i.e., different disease entities) even under the umbrella of the same clinical phenotype. Classification systems and nomenclature should reflect not only superficial clinical differences but more meaningful underlying drivers of those differences.

## Conclusions

In this paper, we review the clinical, etiological, and pathophysiological heterogeneity in ET, and we put forth the argument that our terminology should evolve to reflect and keep pace with advances in science and that “the essential tremors” is a more scientifically appropriate term.

## Author Contributions

EL conceptualized and wrote this paper.

## Conflict of Interest

The author declares that the research was conducted in the absence of any commercial or financial relationships that could be construed as a potential conflict of interest.
